# Measuring Physical Function Capacity in Persons With Haemophilia: A Systematic Review of Performance‐Based Methods

**DOI:** 10.1111/hae.70081

**Published:** 2025-07-20

**Authors:** Catherine Holdsworth, Melanie Bladen, Hannah Harbidge, Wendy Drechsler, Ryan Mahaffey, Sofia Perez‐Alenda, Fionnuala Sayers, Karen Strike, Merel Timmer, David Stephensen

**Affiliations:** ^1^ University Hospitals Dorset, Bournemouth UK/Southern Haemophilia Network Poole UK; ^2^ Great Ormond Street Hospital for Children NHS Foundation Trust London UK; ^3^ East Kent Hospitals University NHS Foundation Trust Canterbury UK; ^4^ Faculty of Sport, Technology and Health Sciences St Mary's University London UK; ^5^ Faculty of Physiotherapy University of Valencia Valencia Spain; ^6^ Belfast City Hospital Belfast UK; ^7^ Faculty of Health Sciences Rehabilitation Science McMaster Children's Hospital Hamilton Health Sciences McMaster University Hamilton Canada; ^8^ University Medical Center van Creveldkliniek Utrect the Netherlands; ^9^ Faculty of Medicine Health and Social Care Canterbury Christ Church University Canterbury UK

**Keywords:** core outcome set, haemophilia, joint health, musculoskeletal, physical capacity, physical function, systematic review

## Abstract

**Introduction:**

Currently, physical health assessments in persons with haemophilia focus on bleed‐related events and after‐effects. The aim of the systematic review was to review and apply standardised criteria to evaluate reliability, responsiveness and construct validity of performance‐based instruments evaluating physical capability in persons with haemophilia.

**Methods:**

Medline, CINAHL, Embase, EMCARE, and Cochrane (inception‐March 2024) were searched using COSMIN filters for 7 performance‐based tests in haemophilia, supplemented by manual searches. Reliability, responsiveness and construct validity of the six‐minute walk test (6MWT), timed up and go test (TUG), timed up and down stairs (TUDS), 30‐second sit‐to‐stand (30‐STS), single leg stance (SLS), tandem stance (TS) and single hop for distance (SH) were evaluated.

**Results:**

The search yielded 88 abstracts; 25 studies remained after full‐text screening, covering 5 of 7 performance‐based instruments: 6MWT, TUG, TUDS, SLS, and 30‐STS. No performance‐based test was evaluated for all properties across all ages. Only TUG in adults and older adults and 6MWT in children and adolescents has been tested for all properties. No test received a high grading. Low and very low grades were given mostly for indeterminate results, small or single studies and lack of a similar construct of comparator. The 6MWT in all age groups was the only performance‐based test graded moderate, and this was for responsiveness.

**Conclusion:**

With increasing use of performance‐based methods of physical function capacity, evaluating measurement properties is a priority. Until evidence is generated, we can only advocate the 6MWT to monitor responsiveness in adult persons with haemophilia affected with marked arthropathy.

**Summary:**

**Understanding Physical Health in People with Haemophilia**

Currently, when we check the physical health of people with haemophilia, we mostly look at problems caused by bleeding. But we wanted to see if there are better ways to measure how well people with haemophilia can move and do daily activities.

**What We Did**

We looked through a lot of medical studies (up to March 2024) to find information on 7 specific physical tests. These tests measure things like:

- How far someone can walk in six minutes (6‐minute walk test or 6MWT)
- How long it takes to stand up, walk a short distance, and sit down (Timed Up and Go test or TUG)
- How long it takes to go up and down stairs (timed up and down stairs or TUDS)
- How many times someone can stand up from a chair in 30 seconds (30‐second sit‐to‐stand or 30‐STS)
- How long someone can stand on one leg (single leg stance or SLS)
- How long someone can stand with one foot directly in front of the other (Tandem Stance or TS)
- How far someone can hop on one leg (single hop for distance or SH)

We wanted to see how reliable (consistent), responsive (can detect changes), and valid (measures what it's supposed to) these tests were for people with haemophilia.

**What We Found**

We found 25 studies that looked at 5 of the 7 tests (6MWT, TUG, TUDS, SLS, and 30‐STS).

Here's what stood out:

- No single test was good for everything and for all ages.
- Only the TUG test (for adults) and the 6MWT (for children) had been fully studied for all aspects (reliability, responsiveness, and validity).
- None of the tests were rated as highly effective overall. Most got low ratings because the results weren't clear, studies were small, or there wasn't enough good information to compare them to.
- The 6MWT was the only test that received a ‘moderate‘ rating, but only for its ability to show changes in all age groups.

**What This Means**

It's becoming more common to use these kinds of physical tests to understand how well people function. So, it's very important to know if these tests work well for people with haemophilia.

For now, based on the evidence, we can only suggest using the 6‐minute walk test (6MWT) to track how much someone's physical ability changes over time, especially for adults with haemophilia who have severe joint problems. We need more research to find better tests.

## 1 Introduction

Haemophilia care is entering a transformative phase with the advent of potentially life‐changing treatments. These new therapies aim to achieve zero bleeds and prevent joint damage. Early prophylaxis in children and young people can prevent or minimize arthropathy, with the goal of a bleed‐free life [1]. Meanwhile, adults with existing joint arthropathy may experience stabilisation or a slower decline in their physical health [2]. The current assessment of musculoskeletal and physical health in haemophilia focuses on bleed‐related events and their after‐effects. This includes the frequency of bleeds, pain, body structure and function, as well as self‐reported measures of activity and participation [3]. In their review of alternative outcomes to bleeding rate, Castaman and colleagues [4] suggested combining physical examination with imaging techniques that assess joint structures might offer a more effective monitoring approach in the context of new therapies. Furthermore, they recommended that combining these with patient‐reported outcomes would be ideal.

The World Health Organization's International Classification of Functioning (ICF) views health as the result of interactions between body structure and function, activities, participation, and personal and environmental factors. To obtain a comprehensive assessment of a person's health, it is recommended to evaluate all ICF domains by combining objective and self‐reported measurement tools. The ICF defines ‘activity’ as ‘the execution of a task or action by an individual’ [5]. Within the ICF Activities domain, two qualifiers are proposed: performance and capacity. The performance qualifier describes what an individual does in his or her current environment. Since the current environment always includes the overall societal context, performance can also be understood as ‘involvement in a life situation’ or ‘the lived experience’ of people in their actual context. The capacity qualifier describes an individual's ability to execute a task or an action. This construct indicates the highest probable level of functioning of a person in each domain at a given moment. For a full understanding of health, both performance and capacity constructs should be evaluated [5].

There have been three editions of the WFH guidelines for the management of haemophilia with the most recent published in 2020 [3], with increasing discussion and importance on monitoring health status and outcome. With regards to the performance construct of the ICF Activities domain, the self‐report Hemophilia Activities List (HAL) and paediatric Hemophilia Activities List (pedHAL), recommended by the World Federation of Haemophilia (WFH) [3], evaluate an individuals’ perception of their ‘lived experience’ of the performance of tasks in his or her current environment. With regards to the capacity construct of the ICF Activities domain, the Functional Independence Score in Hemophilia (FISH) is a haemophilia‐specific performance‐based tool measuring an individual's independence in performing activities of daily living, transfers and mobility. It evaluates the capability of eight activities in three categories: self‐care, transfers and locomotion, with each activity scored according to the amount of assistance required to perform the task [6]. Due to the ceiling effect of the FISH in people with little arthropathy, it is recommended for populations with more advanced joint disease [7].

As a first step to identify appropriate outcome instruments that evaluate the capability construct of the Activities domain of the ICF, that is, an individual's ability to execute a task or an action, we recently identified seven performance‐based instruments evaluating capability that physiotherapists considered practical in the clinical setting [8]. Utilising a consensus‐based, decision analysis approach, a scoping review and a two‐round international DELPHI study, the capability instruments identified were the 6‐minute walk test (6MWT), timed up and down stairs (TUDS), 30‐second sit‐to‐stand (30‐STS), single leg stance (SLS), tandem stance (TS), single hop for distance (SH), and timed up and go test (TUG) [8]. To enable accurate interpretations of treatment effects and facilitate evidence‐based decision‐making, a thorough evaluation of the measurement properties of these instruments is essential.

Building on the previous work, the aim of this study is to systematically review the published literature and apply a standardised set of criteria to evaluate the reliability, responsiveness and construct validity of the seven performance‐based instruments in people with haemophilia. The results will provide clinicians and researchers with a basis for choosing a performance‐based method to measure capability for clinical practice or for a research study.

## 2 Methods

The systematic review was undertaken according to the COnsensus‐based Standards for the selection of health Measurement INstruments (COSMIN) [9, 10, 11, 12] and the protocol is registered with the International Prospective Register of Systematic Reviews PROSPERO (CRD42024445368). Covidence systematic review software (Veritas Health Innovation, Melbourne, Australia. www.covidence.org) was used to manage article selection, data extraction and quality assessment.

### 2.1 Study Group

The study group was established in 2017 to identify performance‐based instruments of physical ability and function for monitoring musculoskeletal health in people with haemophilia. Members were invited based on their international standing in paediatric and/or adult haemophilia clinical practice and/or research and/or expertise in outcome measurement and included representatives from Canada, the Netherlands, Spain and the UK. In 2023, the group published the results of the international DELPHI study, identifying the performance instruments evaluated in this systematic review [8].

### 2.2 Literature Search

This systematic review is a focused subset of a larger review on musculoskeletal conditions, presenting only the methods and results relevant to the haemophilia population.

CH searched the following databases using the COSMIN validated filter to find studies on measurement properties: Medline, CINAHL, Embase, EMCARE and Cochrane databases, from database inception to 31 March 2024. The full search strategy is listed in Supplementary File 1. In short, in accordance with the PICO format [13], a combination of different variations of the following text words was used: Population: haemophilia; Intervention: the seven performance‐based tests (6MWT, TUDS, 30STS, SLS, TS, SH, TUG); and Outcome: validity, reliability, measurement error, hypothesis testing or responsiveness; we did not use a comparator. Additional articles were identified by manually searching references of the retrieved articles and published abstracts from the WFH and European Association of Haemophilia and Allied Disorders (EAHAD) congresses since the year 2000. We excluded case reports and letters. Non‐English articles were excluded.

### 2.3 Selection of Articles

Articles identified from the search were imported into the online tool Covidence. After duplicates were removed, abstracts and titles were independently screened by C.H. and one other of the team of nine reviewers according to the PICO inclusion criteria. All reviewers were blinded. Conflicts were resolved with discussion by M.B. and D.S. The full‐text article was retrieved from all abstracts that fulfilled the inclusion criteria. The retrieved articles were independently reviewed again by C.H. and one other of the team of reviewers (each blinded, and conflicts resolved with discussion by M.B. and D.S.) against the inclusion criteria and included if the criteria were met and included appropriate statistical analysis outlined in the COSMIN guidelines [9, 10, 11, 12], described below.

Reliability: the degree to which the measurement is free from error, reported as an interclass correlation coefficient (ICC), weighted kappa, or Pearson correlation coefficient.

Measurement error: the precision of the instrument reported as standard error of measurement (SEM), minimal important change (MIC), minimal clinically important difference (MCID), smallest detectable change (SDC) or limits of agreement (LoA).

Responsiveness: the degree to which the instrument can detect change that is likely due to an intervention or where there has been a change over time reported as an effect size or mean difference with confidence intervals. Measurement of effect size falls under 2 general categories: Group difference indices which estimate the magnitude of difference between two or more groups are reported as standardised mean difference (SMD), effect size median (ESM), Cohen's *d*, Hedges *g* or Strength of association indices which estimate the magnitude of shared variance between two or more variables are reported as omega squared (*ω* ^2^), Eta squared (*η* ^2^), Epsilon squared (*ε* ^2^), and Kendall's *W* when calculated from the Friedman test.

Construct Validity: the extent to which scores on a particular instrument relate to other measures in a manner that is consistent with theoretically derived hypotheses concerning the concepts that are being measured, reported as a measure of correlation, Pearson's (*r*) or Spearman's rank (rho), or area under the curve (AUC).

A comprehensive guideline and flowchart were developed for each stage of the screening process, and the review team all attended a training session prior to screening (Supplementary File 2). All reviewers participated in a validation process, independently reviewing the same set of 25 randomly selected abstracts and 10 randomly selected full‐text articles. This was completed prior to screening. Reliability metrics for the validation exercises were an absolute agreement of 96% for abstracts and 89% for full‐text, and a Cohen's kappa of 0.60 and 0.58, respectively.

### 2.4 Data Extraction

A description of the performance‐based method was extracted from the included articles, along with details of participants, study design, and information on the measurement properties of the instruments using a checklist based on standardised Covidence templates. Data included the construct of interest of the studies, population of interest and the measurement instruments used, and publication details: number of participants, demographic information (including gender, age, and country), haemophilia type (A/B and severity), test name, brief description of the test, health professional completing the test and any training undertaken. Joint health status (Hemophilia Joint Health Score [HJHS]) [14] was also collected. The HJHS is a tool used to assess joint health in people with haemophilia, evaluating the impact of bleeding on the elbow, knee, and ankle joints by considering factors such as range of motion, pain, and swelling. Data were independently extracted by two reviewers (M.B. and D.S.), with consensus resolved with discussion.

### 2.5 Data Synthesis

Evidence of reliability, measurement error, responsiveness and construct validity were pooled, and data summarised according to the criteria described in Table 1 [12].

**TABLE 1:** Criteria for summarising evidence of construct validity, reliability, measurement error and responsiveness [9, 10, 11, 12].

	Positive (+)	Indeterminate (?)	Negative (−)
**Construct validity**	Correlation with an instrument measuring the same construct ≥ 0.50	Solely correlations determined with unrelated constructs	Correlation with an instrument measuring the same construct < 0.50
**Test‐retest reliability**	ICC/weighted kappa ≥ 0.70, or Pearson's *r* ≥ 0.80	Neither ICC/weighted kappa, nor Pearson's *r* determined	ICC/weighted kappa < 0.70, or Pearson's *r* < 0.80
**Measurement error**	MIC or SEM < SDC, or MIC outside the LOA, or SEM < SD	MIC or SEM not defined	MIC or SEM > SDC, or MIC equals or is inside LOA, or SEM > SD
**Responsiveness**	Results are in accordance with the hypotheses and/or medium or large effect size, and/or confidence interval do not include zero	No hypothesis defined by authors or research team and/or small effect size	Results are not in accordance with the hypotheses and/or effect size is very small, and/or confidence intervals include zero

*Note*: SDC = 1.96 × √2 × SEM.

Abbreviations: ICC = intraclass coefficient, MIC = minimal important change, *r* = correlation coefficient, SD = standard deviation, SDC = standard difference change, SEM = standard error of measurement, LOA = levels of agreement.

Data were reported in the following age categories [7]: younger children (4–10 years), adolescents (11–17 years), adults (18–54 years) and older adults (55+ years).

### 2.6 Quality Assessment—Risk of Bias

A quality risk of bias assessment was undertaken for all included studies using COSMIN criteria, described below [12]. Each criterion was assessed as very good, adequate, doubtful or inadequate. For full criteria see Supplementary File 2. Blinded quality assessment was independently extracted by 2 reviewers (M.B. and D.S.), with consensus resolved with discussion.

Reliability/ measurement error: Stability of participants (participants condition unchanged), appropriateness of time interval (too long and condition may change, too short and participants might recall results), similarity of test conditions (condition consistent for each repeated measurement), assessor blinding (unaware of previous results), and preferred statistical method (Table 1).

Responsiveness: An adequate description was provided of all important characteristics of the groups/subgroups (baseline characteristics clearly described), the preferred statistical method, and the study design (Table 1).

Construct validity: similarity of the construct of the comparator instrument (same underlying concept), preferred statistical method, and study design (Table 1).

A best evidence synthesis was performed when there were results from multiple studies using the same performance test. The possible levels of evidence for a measurement property are ‘strong,’ ‘moderate,’ ‘limited,’ ‘conflicting,’ or ‘unknown.’ Best evidence synthesis was given an overall grade according to the GRADE criteria: high, moderate, low and very low, using the level of evidence, quality assessment of studies as well as the number of related studies evaluating each measurement property [40]. According to the GRADE system, outcomes start on ‘high’ quality, after which they may be downgraded one level per criteria if it is deemed to have a serious risk (−1) or very serious risk (−2).

## 3 Results

**FIGURE 1:**
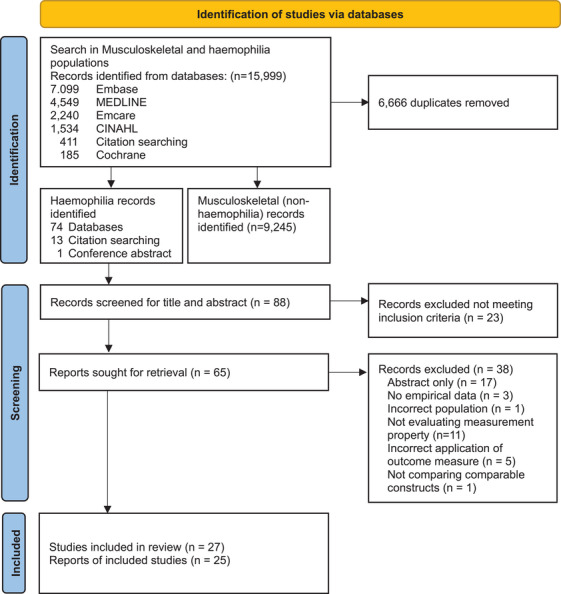
PRISMA flow diagram depicting number of records identified, included and excluded.

We obtained 88 abstracts from the searches, and all were screened against the inclusion criteria (Figure 1). For completeness, Figure 1 also includes search results for the larger review on musculoskeletal conditions (*n* = 15,999). Sixty‐five abstracts met the inclusion criteria and were selected for further inspection of the full‐text article. Following full‐text review, 38 articles did not meet the inclusion criteria. The most common reasons for excluding a study were conference abstract only and not evaluating a measurement property. Twenty‐seven studies were initially included, containing two systematic reviews with meta‐analyses [41, 42]. Full text of three relevant studies (data on one of the seven outcome measures) from the systematic reviews were retrieved for full text review—two were already included in the data extraction, and a third did not include the relevant statistical approach for inclusion. The meta‐analyses were not included. Finally, twenty‐five studies were included (Table 2), referring to five out of the seven performance‐based tests: TUG, 6MWT, SLS, TUDS, and 30STS. No study met the inclusion criteria for TS or SH. Inter‐rater reliability (Cohen's kappa) for reviewers was 0.61 for abstract screening and 0.64 for full‐text screening, indicating good inter‐rater reliability (Cohen's kappa > 0.60).

**TABLE 2:** Characteristics of included studies.

			Patient population					
Study Reference	Country of study	Measurement property assessed	Sample size	Haemophilia type, severity and treatment characteristics	Age (years)	Baseline HJHS	Test description	Assessor performing measurement
**TUG**								
Calatayud et al. [15]	Spain	Reproducibility Responsiveness	Total n = 20; 10 PWH in each group	SHA/B *n* = 17; MHA/B *n* = 1; MIHA/B *n* = 2; HA *n* = 18; HB *n* = 2; No patients with inhibitor; Prophylaxis *n* = 17	Exercise:36.3 ± 10.5^b^ Usual care: 39.1 ± 8.4^b^	Not reported	Performed over 3‐m, able to use arms, shod or barefoot not reported. TUG repeated and best time used.	Profession or training of assessor not reported
Chantrain et al. [16]	Belgium	Construct validity	Total PWH *n* = 40 Missing data *n* = 3	SMHA/B *n* = 17; SH = 14; MiHA/B (*n* = 23); No patients with inhibitor; HA *n* = 26; HB *n* = 14; Prophylaxis *n* = 16; Gene therapy *n* = 1	SHA: 68 (63–73)^a^ MHA: 68 (62–70)^a^	SMHA/B: 40 (23–57)^a^ MIHA/B: 4 (2–9)^a^	Length of course, use of arms, shod or barefoot, single or multiple performance not reported (refers to another publication)	Profession or training of assessor not reported
Chantrain et al. [17]	Belgium	Construct validity	Total PWH *n* = 104	SHA/B *n* = 77; MHA/B *n* = 27; No patients with inhibitor; HA *n* = 86; HB *n* = 18 Prophylaxis *n* = 84	37.4 ± 13.7	23 ± 19.6^b^	Performed over 3‐m, use of arms, shod or barefoot not reported. TUG repeated and mean time used.	Research fellow Profession or training not reported
Fearn et al. [18]	Australia	Construct validity	Total PWH *n* = 20	SHA/B *n* = 14); MHA/B *n* = 4; MiHA/B *n* = 2; HA *n* = 19; HB *n* = 1; No patients with inhibitors; Prophylaxis *n* = 7; 50% reported fall in previous 12 months Inclusion: able to stand unsupported for longer than 30 s	39.4^b^	Not reported	Performed over 3‐m, use of arms, shod or barefoot, single or multiple performance not reported	Profession or training of assessor not reported
Gonen et al. [19]	Turkey	Responsiveness	Total PWH *n* = 24; 8 in each group 3 withdrawn/ dropped out	SHA *n* = 15; MHA *n* = 6; HA *n* = 24 Patients with inhibitors excluded. Inclusion: target knee joint	Group 1: 11.43 ± 1.81 Group 2: 13.00 ± 3.06 Group 3: 11.14 ± 2.41	Not reported	Performed over 3‐m, use of arms, shod or barefoot not reported. TUG repeated and mean time used.	Physiotherapist. Training not reported
Lobet et al. [20]	Belgium	Construct validity	Total PWH *n* = 130	SHA/B *n* = 80; MHA/B *n* = 29; MiHA/B *n* = 21 HA *n* = 101; HB *n* = 29; Prophylaxis *n* = 89	45 (29–61)^a^	14 (4–37)^a^	Performed over 3‐m, use of arms, shod or barefoot not reported. TUG repeated and mean time used.	Profession or training of assessor not reported
Moreno‐Segura et al. [21]	Spain	Responsiveness	Total PWH *n* = 21; 11 in EX group, 10 in C group 2 lost to follow‐up	SHA *n* = 18; SHB *n* = 1 Inclusion: arthropathy in at least one of Elbow, knee or ankle joints	EX: 45.00 ± 8.56 Control group: 37.89 ± 13.31	EX: 39.70 ± 19.24; Control: 25.44 ± 11.17	Length of course, use of arms, shod or barefoot, single or multiple performance not reported (refers to another publication)	Physiotherapist. Training not reported
Tat et al. [22]	Turkey	Responsiveness	Total PWH *n* = 23; 12 in EX + Manual therapy group, 11 in EX group 6 withdrawn/lost to follow‐up	SHA *n* = 23 Status of prophylaxis and inhibitor not reported Inclusion: arthropathy in any lower limb joint	26 (11)^a^	Total HJHS not reported. Knee joint HJHS EX = 8 (8.5) Control = 11 (7) Ankle joint HJHS Ex = 4 (6) Control = 3 (4.25)	Performed over 3‐m, use of arms, shod or barefoot single or multiple performance not reported.	Physiotherapist. Training not reported
Taylor et al. [23]	United Kingdom	Construct validity	Total PWH *n* = 80	SHA/B *n* = 40; MHA/B *n* = 10; MiHA/B *n* = 30; 1 patient with inhibitor HA *n* = 74; HB *n* = 6 Prophylaxis *n* = 39 Inclusion: able to walk 10‐m	44.5 (32–56)	5 (0–19.5)^a^	Performed over 3‐m, able to use arms, shod or barefoot not reported. Single TUG performance.	Physiotherapist. Training not reported
Tomschi et al. [24]	Germany	Construct validity	Total PWH *n* = 29 Missing data *n* = 1	SHA/B *n* = 26; MHA/B *n* = 3 HA *n* = 23; HB *n* = 6; Prophylaxis *n* = 27	57.0 (48.0–61.5)^a^	HJHS = 35 (23–50)^a^	Performed over 3‐m, use of arms, shod or barefoot single or multiple performance not reported.	Profession or training of assessor not reported
Uzuner et al. [25]	Turkey	Construct validity	Total *n* = 56 PWH: *n* = 30 Control: *n* = 26	Sedentary adult males with SHA (*n* = 30), presence of inhibitor excluded. Inclusion: sedentary PWH Knee involved in all patients, elbow/ ankle involvement in 28 patients.	PWH: 35.40 ± 9.44^b^ Control: 32.78 ± 6.00^b^	Not reported	Performed over 3‐m, use of arms, shod or barefoot not reported. TUG repeated and best time used.	Profession or training of assessor not reported
Van Genderen et al. [26]	The Netherlands	Construct validity	Total PWH *n* = 127	SHA *n* = 110; SHB *n* = 17 Status of prophylaxis and inhibitor not reported	42 (31–51)^a^	Not reported	Performed over 3‐m, use of arms, shod or barefoot, single or multiple performance not reported.	Physiotherapist. Training not reported
**6MWT**								
Atay et al. [27]	Turkey	Responsiveness	Total PWH *n* = 29; 14 supervised exercise, 15 unsupervised exercise, 5 dropped out/withdrawn	HA *n* = 25; HB *n* = 4 SHA/B *n* = 24; MHA/B *n* = 5	EX supervised: 14.86 ± 2.07^b^ EX unsupervised: 13.61 ± 3.12^b^	Supervised Ex = 11.29 ± 8.32^b^; Unsupervised Ex = 13.11 ± 11.10^b^	Length of course (refers to another publication), shod or barefoot, single or multiple performance not reported.	Physiotherapist. Training not reported
Azab et al. [28]	Saudi Arabia	Responsiveness	Total PWH *n* = 56; 28 received usual care, 28 virtual reality EX plus usual care 4 lost to follow‐up	MHA *n* = 56 Prophylaxis *n* = 56 Inclusion: ≥ 4 bilateral knee haemarthrosis in the previous 6 months	Usual care:12.89 ± 1.19^b^ EX: 12.46 ± 1.50^b^	Not reported	Performed over 50‐m, Shod or barefoot not reported. Single performance of 6MTW	Profession or training of assessor not reported
Bladen et al. [29]	United Kingdom	Responsiveness	Total PWH *n* = 9; 5 in EX group, 4 in Usual care group	SHA *n* = 7; SHB *n* = 2 Prophylaxis *n* = 9	9.77 ± 2.18^b^	Not reported	Performed over 10‐m, shod or barefoot not reported. Single performance of 6MTW	Physiotherapist. Training not reported
Cuesta‐Barriuso et al. [30]	Spain	Responsiveness	Total PWH *n* = 65; 33 fascial therapy, 32 usual activities. Withdrawals/lost to follow‐up *n* = 3	HA *n* = 42; HB *n* = 23 SHA/B *n* = 55; MiHA/B *n* = 10; inhibitor *n* = 5 Prophylaxis *n* = 49 Inclusion: Ankle arthropathy	Fascial therapy: 40.8 ± 9.2^b^ Usual activities: 35.6 ± 12.5^b^	Ankle Joint Fascial therapy: Right Ankle = 7.3±3.4^b^, Left Ankle = 7.1± 3.5^b^ Usual activities: Right Ankle = 6.4 ± 3.0^b^, Left Ankle = 7.0 ± 3.1^b^	Performed over 20‐m, Shod or barefoot not reported. Single performance of 6MTW.	Physiotherapist. Training not reported
Deniz et al. [31]	Turkey	Responsiveness	Total PWH *n* = 20; 10 EX group, 9 control group Withdrawals *n* = 1	HA *n* = 12; HB (*n* = 7) SHA/B *n* = 12; MHA/B *n* = 7 Prophylaxis *n* = 12 Inclusion: arthropathy of knee or ankle joint	26.3 ± 7.1^b^	EX: 20.3 ± 4.2^b^; Control: 15.9 ± 8.9^b^	Performed over 20‐m. Shod or barefoot, single or multiple performance not reported.	Physiotherapist. Training not reported
Elnaggar et al. [32]	Saudi Arabia	Responsiveness	Total PWH *n* = 48; 24 in hydro‐kinesiotherapy EX group and 24 in usual EX group Missing data *n* = 3	MHA *n* = 48 Prophylaxis *n* = 48 Inclusion: ≥ 4 unilateral knee haemarthrosis in the previous 6 months with ≥ Grade 3 muscle strength	EX Group 1: 13.17 ± 2.20^b^ EX group 2: 12.88 ± 2.52^b^	Not reported	Performed over 30‐m. Shod or barefoot, single or multiple performance not reported.	Profession or training of assessor not reported
El‐Shamy et al. [33]	Saudia Arabia	Responsiveness	Total PWH *n* = 30; 15 in usual care and 15 in usual care + whole body vibration therapy	HA *n* = 30 Haemophilia severity not reported. Prophylaxis *n* = 30 Inclusion: bilateral haemarthrosis	Usual care: 11.90 ± 2.74^b^ Whole body vibration: 12.22 ± 2.33^b^	Not reported	Performed over 20‐m. Shod or barefoot. Single performance of 6MTW	Profession or training of assessor not reported
Kennedy et al. [34]	Ireland	Construct validity	Total PWH *n* = 53; *n* = 45 included in validity analysis	SHA *n* = 31; SHB *n* = 15; MHA/B *n* = 7 5 persons with an inhibitor Prophylaxis *n* = 47	44 (33–51)^a^for *n* = 53 cohort	29 (20–34)^a^ for *n* = 53 cohort	Performed over 30‐m (refers to another publication). Shod or barefoot, single or multiple performance not reported.	Profession or training of assessor not reported
Radzevia et al. [35]	Lithuania	Construct validity	Total PWH *n* = 24	SHA/B *n* = 21; MiHA/B *n* = 3 HA *n* = 21; HB *n* = 3 Prophylaxis *n* = 21	12.58 ± 3.01^b^	18.46 ± 7.28^b^	Walkway distance, shod or barefoot, single or multiple performance not reported (refers to another publication suggesting 20‐m)	Profession or training of assessor not reported
Stephensen et al. [36]	United Kingdom	Reproducibility	Total PWH *n* = 21; *n* = 8 included in reliability analysis	SHA *n* = 20; SHB *n* = 1 Prophylaxis *n* = 21 Inclusion: experienced at least one lower limb joint bleed since birth	9.16 ± 1.94^b^	Not reported	Performed over 15‐m with shoes. Single performance of 6MTW.	Profession or training of assessor not reported
**SLS**								
Czepa et al. [37]	Germany	Construct validity	PWH *n* = 48; non‐haemophilia *n* = 43 Both grouped together for validity analysis Missing data *n* = 10 (unable to perform test)	SHA/B *n* = 45; severity of 3 not reported HA *n* = 46; HB *n* = 2; 4 persons with an inhibitor Prophylaxis *n* = 26	44 (11)^a^ 42 (11)^a^	Not reported OJS = 29.1 ± 9.8^b^	Performed with eyes open and barefoot. Maximum time limited to 30 s. SLS repeated and mean time used. Arm position not reported	Profession or training of assessor not reported
Runkel et al. [38]	Germany	Responsiveness	Total PWH n = 64; 32 in EX group and 32 in usual care group, Withdrawals/missing data *n* = 12	SH *n* = 59; MH *n* = 5; type A/B not reported; 1 person with an inhibitor Prophylaxis 92% in EX group and 86% usual care group Inclusion: no previous fitness training	EX: 41.9 ± 10.6^b^ Usual care: 40.3 ± 8.8^b^	Not reported Gilbert Score EX = 18.4 ± 6.1 Usual care: 16.5 ± 6.2	Test procedure refers to another publication. Maximum time limited to 30 s.	Profession or training of assessor not reported
Stephensen et al. [36]	United Kingdom	Reproducibility	Total PWH *n* = 21; *n* = 8 included in reliability analysis	SHA *n* = 20; SHB *n* = 1 Prophylaxis *n* = 21 Inclusion: experienced at least one lower limb joint bleed since birth	9.16 ± 1.94^b^	Not reported	Performed with eyes open, barefoot and hands on hips. No time limit. SLS repeated and best time used.	Profession or training of assessor not reported
Tomschi et al. [24]	Germany	Construct validity	Total PWH *n* = 29 Missing data *n* = 1	SHA/B *n* = 26; MHA/B *n* = 3 HA *n* = 23; HB *n* = 6; Prophylaxis *n* = 27	57.0 (48.0–61.5)^a^	HJHS = 35 (23–50)^a^	Performed with eyes open and closed. Maximum time limited to 45 s. SLS repeated and mean time used. Arm position, shod or barefoot not reported.	Profession or training of assessor not reported
**TUDS**								
Bladen et al. [29]	United Kingdom	Responsiveness	Total PWH *n* = 9; 5 in EX group and 4 in Usual care group	SHA *n* = 7; SHB *n* = 2 Prophylaxis *n* = 9	9.77 ± 2.18^b^	Not reported	Number of steps 12. Step height, shod or barefoot not reported. Test repeated and best time used.	Physiotherapist. Training not reported
Stephensen et al. [36]	United Kingdom	Reproducibility	Total PWH *n* = 21; *n* = 8 included in reliability analysis	SHA *n* = 20; SHB *n* = 1 Prophylaxis *n* = 21 Inclusion: experienced at least one lower limb joint bleed since birth	9.16 ± 1.94^b^	Not reported	Number of steps 12. Step height, shod or barefoot not reported. Test repeated and best time used.	Profession or training of assessor not reported
**30secSTS**								
Cruz‐Montecinos et al. [39]	Chile	Construct validity Responsiveness	Total *n* = 32; Haem group = 17, Control group = 15	SHA/B *n* = 15; MHA/B *n* = 2 HA *n* = 15; HB *n* = 2 Prophylaxis and inhibitor status not reported. Inclusion: Sedentary PWH	36.4 ± 10.9 34.3 ± 14.6	41^a^	Chair height 43 cm. Use of arms, shod or barefoot not reported. Test repeated and best number used.	Profession or training of assessor not reported

Abbreviations: 6MWT = six‐minute walk test, 30secSTS = 30‐second sit to stand, EX = exercise, HA = haemophilia A, HB = haemophilia B, HJHS = haemophilia joint health score, MHA = moderate haemophilia A, MiHA = mild haemophilia A, OJS = orthopaedic joint score; PWH = person with haemophilia, SHA = severe haemophilia A, SLS = single leg stand, TUG = timed up and go, TUDS = timed up and down stairs.

^a^ Median and interquartile range.

^b^ Mean ± standard deviation.

Description of measurement properties from included studies is reported in Table 3 with a summary provided in Table 4. Quality assessment for each included study is reported in Table 4, with a synthesis of evidence provided in Table 5.

**TABLE 3:** Description of measurement properties evaluated.

	Study population	Reproducibility		Construct validity		Responsiveness				
Study reference	Age group	Design	Main results	Hypothesis reported	Main results	Design	Hypothesis reported	Treatment/group	Comparator/group	Main results
**TUG**										
Calatayud et al. [15]	Adults Older adults	Measurement error	MCID = 0.3	**—**	**—**	RCT	Yes	Exercise: Progressive moderate‐vigorous elastic resistance	Usual care	MD (95% CI) = 0.63 (0.18 to 1.09) Cohen's *d* ES = 0.5
Chantrain et al. [16]	Older adults	**—**	**—**	Yes	Pain Severity: *r*_s_ = 0.441 Pain interference: *r*_s_ = 0.607	**—**	**—**	**—**	**—**	**—**
Chantrain et al. [17]	Adults Older adults	**—**	**—**	Yes	HAL complex lower limb score: *r*_s_ = 0.353	**—**	**—**	**—**	**—**	**—**
Fearn et al. [18]	Adults Older adults	**—**	**—**	No	Walking speed: *r*_p_ = 0.424 Step width: *r*_p_ = 0.311 Turning sway: *r*_p_ = 0.530	**—**	**—**	**—**	**—**	**—**
Gönen et al. [19]	Children Adolescents Adults	**—**	**—**	**—**	**—**	RCT	Yes	Closed Kinetic Chain Exercise (CKEx)	1) Conventional exercise (CEx) 2) Control (C)	Cohen's *d* ES: CKCEx versus CEx: d = 0.56 CKCEx versus Control: *d* = 0.83 CEx versus Control: *d* = 0.33
Lobet et al. [20]	Adults Older adults	**—**	**—**	No	ACTIVLIM‐Hemo: *r*_s_ = −0.501 HAL: *r*_s_ = −0.383	**—**	**—**	**—**	**—**	**—**
Moreno‐Segura et al. [21]	Adults Older adults	**—**	**—**	**—**	**—**	Non‐RCT	Yes	Exercise: Combined therapeutic exercise and CBT	Usual activities	End of intervention: MD (95%CI) = 0.07 (−0.20 to 4.41) 12 weeks post intervention: MD (95% CI) = 0.09 (−0.30 to 4.06)
Tat et al. [22]	Adults	**—**	**—**	**—**	**—**	RCT	No	Manual therapy	Control group	ES = 0.19
Taylor et al. [23]	Adults Older adults	**—**	**—**	No	FSST All: *r*_p_ = 0.753; SHA/B: *r*_p_ = 0.770; MiHA/B: *r*_p_ = 0.783	**—**	**—**	**—**	**—**	**—**
Tomschi et al. [24]	Adults Older adults	**—**	**—**	No	HJHS *r*_s_ = 0.647; HEP‐TEST‐Q *r*_s_ = 0.466; TSK‐11 *r*_s_ = 0.344 FES‐1 *r*_s_ = 0.393	**—**	**—**	**—**	**—**	**—**
Uzuner et al. [25]	Adults	**—**	**—**	No	HJHS LL: *r*_s_ = −0.483; Pain (VAS): *r*_s_ = −0.416; Fear of movement: *r*_s_ = −0.580	**—**	**—**	**—**	**—**	**—**
Van Genderen et al. [26]	Adults Older adults	**—**	**—**	No	HAL Sum: *r*_s_ = 0.59 HAL Lower limb basic: *r*_s_ = 0.55 HAL Lower Limb complex *r*_s_ = 0.62	**—**	**—**	**—**	**—**	**—**
**6MWT**										
Atay et al. [27]	Children Adolescents	**—**	**—**	**—**	**—**	RCT	Yes	Supervised exercise: Individual	Individual counselling unsupervised home‐exercise program	Cohen's *d* ES = 0.551
Azab et al. [28]	Children Adolescents	**—**	**—**	**—**	**—**	RCT	No	Exercise–virtual reality	Exercise–traditional physiotherapy	Partial *η* ^2^ ES = 0.17
Bladen et al. [29]	Children Adolescents	—	—	—	—	RCT		Exercise	Usual care	MD (95% CI) = 61.2 (12.5 to 110)
Cuesta‐Barriuso et al. [30]	Adults Older adults	**—**	**—**	**—**	**—**	RCT	No	Fascial therapy	Usual activities	Partial *η* ^2^ ES = 0.08
Deniz et al. [31]	Adults Older adults	**—**	**—**	**—**	**—**	Non‐RCT	Yes	Exercise	Control	End of intervention: Cohen's *d* ES = 1.8 (0.7 to 2.8) 6‐months after intervention: Cohen's *d* ES = 1.3 (0.3 to 2.8)
Elnaggar et al. [32]	Children Adolescents	**—**	**—**	**—**	**—**	RCT	Yes	Plyometric‐based hydro‐kinesiotherapy	Standard exercise program	Partial *η* ^2^ ES = 0.19
El‐Shamy et al. [33]	Children Adolescents	**—**	**—**	**—**	**—**	RCT	No	Conventional Physical Therapy PLUS Whole‐Body Vibration	Conventional Physical Therapy	Cohen's *d* ES = 1.08
Kennedy et al. [34]	Adults Older adults	**—**	**—**	No	HJHS: *r*_p_ = −0.14	**—**	**—**	**—**	**—**	**—**
Radzevič et al. [35]	Children Adolescents	**—**	**—**	No	HJHS: *r*_p_ = −0.938; HAL: *r*_p_ = 0.903	**—**	**—**	**—**	**—**	**—**
Stephensen et al. [36]	Children Adolescents	Between session Test‐retest Measurement error	ICC = 0.78 SEM = 25.22	**—**	**—**	**—**	**—**	**—**	**—**	**—**
**SLS**										
Czepa et al. [37]	Adults Older adults	**—**	**—**	Yes	HEP‐Test‐Q and Left Leg: *r*_s_ = 0.403 (*n* = 80); HEP‐Test‐Q and Right leg: *r*_s_ = 0.439 (*n* = 83) Haemophilia and non‐haemophilia combined. 10 PWH unable to do test and data not included	**—**	**—**	**—**	**—**	**—**
Runkel et al. [38]	Adults Older adults	**—**	**—**	**—**	**—**	RCT	No	Sports therapy	Usual activities	Partial *η* ^2^ ES Right leg = 0.076 Left leg: value not reported as stated non‐significant
Stephensen et al. [36]	Children Adolescents	Within session Test‐retest Measurement error Between session Test‐retest Measurement error	ICC = 0.78 SEM = 20.35 ICC = 0.85 SEM = 15.45	**—**	**—**	**—**	**—**	**—**	**—**	**—**
Tomschi et al. [24]	Adults Older adults	**—**	**—**	No	Eyes open HJHS *r*_s_ = 0.727; HEP‐ Test‐Q *r*_s_ = 0.596; TSK‐11 *r*_s_ = −0.361; FES‐1 *r*_s_ = −0.467 Eyes closed HJHS *r*_s_ = −0.606; HEP‐ Test *Q r*_s_ = 0.592; TSK‐11 *r*_s_ = −0.232; FES‐1 *r*_s_ = −0.451; Age *r*_s_ = −0.573	**—**	**—**	**—**	**—**	**—**
**TUDS**										
Bladen et al. [29]	Children Adolescents	**—**	**—**	**—**	**—**	RCT	No	Exercise	Usual care	MD (95% CI) = −1.73 (−2.51 to −0.94)
Stephensen et al. [36]	Children Adolescents	Within session Test‐retest Measurement error Between session Test‐retest Measurement error	ICC = 0.87 SEM = 1.03 ICC = 0.91 SEM = 0.92	**—**	**—**	**—**	**—**	**—**	**—**	**—**
**30 second STS**										
Cruz‐Montecinos et al. [39]	Adults Older adults	**—**	**—**	Yes	HJHS LL: *r*_s_ = −0.483	Case control	Yes	Haemophilia	Healthy volunteers	Cohen's *d* ES = 1.69

*Note*: Children = 4–10 years; Adolescent = 11–17 years; Adult = 18–54 years; Older adult = > 54 years. Cohen's *d* effect sizes: small = 0.2 to 0.49; medium = 0.5 to 0.79; large ≥ 0.8

Partial eta squared *η* ^2^ (effect size): small = 0.01 to 0.059; medium = 0.06 to 0.139; large ≥ 0.14.

Abbreviations: CI = confidence interval, ES = effect size; FSST = four square step test, HAL = haemophilia activities list, HEP‐TEST‐Q = haemophilia and exercise project‐test‐questionnaire, HJHS = haemophilia joint health score, ICC = intraclass coefficient, LL = lower limb, MCID = minimum clinically important difference, MD = mean difference; MiHA/B = mild haemophilia A or B, OJS = orthopaedic joint score, PWH = person with haemophilia, *r*_s_ = Spearman's rank correlation coefficient, *r*_p_ = Pearson's correlation coefficient, RCT = randomised controlled trial, SEM = standard error of measurement, SHA/B = severe haemophilia A or B, SMD = standard mean difference, TSK = Tampa scale for Kinesiophobia, *η* ^2^ = eta squared.

**TABLE 4:** Summary of data synthesis and quality risk of bias assessment.

			Overall rating ^a^				Risk of bias assessment ^b^					
			Reproducibility									
			Test‐retest repeatability	Measurement error	Construct validity	Responsiveness	Stability of participants	Time interval	Similarity of Test conditions	Assessor blinding	Comparable Group characteristics	Similar construct of comparator
Children and adolescents	6MWT	Atay et al. [27]				+					Adequate	
		Azab et al. [28]				+					Adequate	
		Bladen et al. [29]				+					Adequate	
		Elnaggar et al. [32]				+					Very good	
		El‐Shamy et al. [33]				+					Adequate	
		Radzevia et al. [35]			+							Doubtful
		Stephensen et al. [36]	+	+			Very good	Very good	Very good	Doubtful		
	SLS	Stephensen et al. [36]	+	+			Very good	Very good	Very good	Doubtful		
	TUDS	Bladen et al. [29]				+					Adequate	
		Stephensen et al. [36]	+	+			Very good	Very good	Very good	Doubtful		
Children, adolescents and adults	TUG	Gonen et al. [19]				+					Doubtful	
Adults	TUG	Tat et al. [22]				?					Doubtful	
		Uzuner et al. [25]			+/−							Doubtful
Adults and older adults	TUG	Calatayud et al. [15]		−		+					Very good	
		Chantrain et al. [17]			−							Adequate
		Fearn et al. [18]			−/+							Very good
		Lobet et al. [20]			−/+							Very good
		Moreno‐Segura et al. [21]				−					Very good	
		Taylor et al. [23]			+							Very good
		Tomschi et al. [24]			−/+							Adequate
		Van Genderen et al. [26]			+							Adequate
	6MWT	Cuesta‐Barriuso et al. [30]				+					Very good	
		Deniz et al. [31]				+					Very good	
		Kennedy et al. [34]			−							Doubtful
	SLS	Czepa et al. [37]			−							Doubtful
		Runkel et al. [38]				−/+					Adequate	
		Tomschi et al. [24]			−/+							Adequate
	30STS	Cruz‐Montecinos et al. [39]			−	+					Very good	Adequate
Older adults	TUG	Chantrain et al. [16]			+/−							Adequate

Abbreviations: 6MWT = six‐minute walk test, 30STS = 30‐second sit‐to‐stand, SLS = single leg stand, TUDS = timed up and down stairs, TUG = timed up and go.

^a^ Overall rating as per criteria in Table 1.

^b^ Very good = evidence provided; adequate = reasons provided to assume standard was met; doubtful = reasons to assume standard not clear; inadequate = no evidence provided. For full criteria see Supplementary File 2.

**TABLE 5:** Summary of overall evidence for measurement properties.

		Reproducibility	Construct validity	Responsiveness
**Timed up and go**	Children and adolescents	Not evaluated	Not evaluated	Very low
	Adults and older adults	Very low	Very low	Very low
**Six‐minute walk test**	Children and adolescents	Low	Low	Moderate
	Adults and older adults	Not evaluated	Very low	Moderate
**Single leg stand**	Children and adolescents	Low	Not evaluated	Not evaluated
	Adults and older adults	Not evaluated	Very Low	Very low
**Timed up and down stairs**	Children and adolescents	Low	Not evaluated	Very low
	Adults and older adults	Not evaluated	Not evaluated	Not evaluated
**30‐second sit‐to‐stand**	Children and adolescents	Not evaluated	Not evaluated	Not evaluated
	Adults and older adults	Not evaluated	Low	Low
**Single hop for distance**	Children and adolescents	Not evaluated	Not evaluated	Not evaluated
	Adults and older adults	Not evaluated	Not evaluated	Not evaluated
**Tandem stance**	Children and adolescents	Not evaluated	Not evaluated	Not evaluated
	Adults and older adults	Not evaluated	Not evaluated	Not evaluated

Of the 25 studies included, seven studies (28%) were conducted in children and adolescents [27, 28, 29, 32, 33, 35, 36], 17 (68%) in adults and older adults [15, 16, 17, 18, 19, 20, 21, 22, 23, 24, 25, 26, 30, 31, 34, 37, 38, 39], and one (4%) in children, adolescents and adults [19]. The total number of participants was 1,132 males and no females. Eighteen (76%) studies included participants with haemophilia A and B [15, 16, 17, 18, 20, 21, 23, 24, 26, 27, 29, 30, 31, 34, 35, 36, 37, 39], six with haemophilia A only [19, 22, 25, 28, 32, 33] one study did not report this information [38]. Seven studies (28%) included only participants with severe disease [21, 22, 25, 26, 29, 36, 37], two (8%) with only moderate disease [28, 32], eight (32%) with severe and moderate disease [15, 16, 18, 20, 23], two (8%) with severe and mild disease [30, 35], five (20%) with severe, moderate and mild disease [15, 16, 18, 20, 23], and one study did not report this information [33]. Three studies reported inclusion of participants with inhibitors [34, 37, 38]. Baseline HJHS status was reported in 13 studies (52%) [16, 17, 20, 21, 22, 23, 24, 27, 30, 31, 34, 35, 39]. Of these, 11 reported total HJHS (mean or median ranging from 5 to 41), and two reported single joint scores ranging from 4 to 11) [22, 30].

### 3.1 TUG

Measurement properties for TUG were reported in 12 studies involving 648 PWH [15, 16, 17, 18, 19, 20, 21, 22, 23, 24, 25, 26]. All studies included adults (18–54 years) or older adults (55+ years), and one included young children (4–10 years) and adolescents (11–17 years) along with adults [19]. One study reported reproducibility and responsiveness in adults and older adults (*n* = 20) with a negative rating for reproducibility and a positive rating for responsiveness [15]. Responsiveness was reported in a further three studies (*n* = 88): one in adults and older adults (*n* = 21), negative rating [21]; one in adults (*n* = 23), indeterminate rating [22]; and one in children, adolescents and adults (*n* = 24), positive rating [19]. Eight studies (*n* = 586) reported construct validity: one in older adults (*n* = 40), indeterminate rating [16]; six in adults and older adults (*n* = 490), one negative [17], three indeterminate [18, 20, 24], two positive ratings [23, 26]; and one in adults (*n* = 56), indeterminate rating [25]. Overall, reproducibility for the TUG was rated as negative, while responsiveness and construct validity were rated as indeterminate. Quality assessment was mostly adequate to very good for studies evaluating the TUG. Most studies involved adults or older adults with severe haemophilia and mean HJHS ranging from 5 to 40, suggesting the presence of considerable joint arthropathy. Overall, the level of evidence for the TUG in PWH is conflicting for all measurement properties.

### 3.2 Six‐Minute Walk Test (6MWT)

Measurement properties for 6MWT were reported in 10 studies involving 355 PWH [27, 28, 29, 30, 31, 32, 33, 34, 35, 36], seven in children and adolescents and three in adults and older adults. One study reported reproducibility in children and adolescents (*n* = 8) with a positive rating [36]. Responsiveness was reported in seven studies (*n* = 257): five in children and adolescents (*n* = 172), all with positive ratings [27, 28, 29, 32, 33, 35]; and 2 in adults and older adults (*n* = 85), both with positive ratings [30, 31]. Two studies (*n* = 69) reported construct validity: one in adults and older adults (*n* = 45) [34], negative rating; one in children and adolescents (*n* = 24), positive rating [35]. Overall, for the 6MWT, reproducibility and construct validity in children and adolescents, and responsiveness in all ages were rated positive. Where HJHS was reported (50% of studies), mean scores ranged between 11 and 29, suggesting the presence of considerable multi‐joint arthropathy, including those involving children (mean HJHS between 11 and 18). Quality assessment ranged from doubtful (assessor blinding and comparable construct) to very good (group characteristics for evaluating responsiveness) for studies evaluating the 6MWT. Overall, the level of evidence for the 6MWT in PWH was strong for responsiveness in all ages and limited for reproducibility and construct validity in children and adolescents (single studies).

### 3.3 SLS

Measurement properties for SLS were reported in four studies involving 162 PWH [24, 36, 37, 38], three in adults and older adults and one in children and adolescents. One study reported reproducibility in children and adolescents (*n* = 8) with a positive rating [36]. Responsiveness was reported in one study of adults and older adults (*n* = 32), with an indeterminate rating [38]. Two studies in adults and older adults reported construct validity (*n* = 120), one with a negative [37] and the other with an indeterminate rating [24]. The study with the negative rating included people without a bleeding disorder as well as those with a bleeding disorder. Overall, for the SLS, reproducibility in children and adolescents was rated positive, and construct validity and responsiveness were indeterminate in adults and older adults. HJHS was reported for one study (25%) with a median of 35, suggesting the presence of considerable multi‐joint arthropathy. Quality assessment ranged from doubtful (assessor blinding and comparable construct) to adequate (group characteristics for evaluating responsiveness) for studies evaluating the SLS. Overall, the level of evidence for the SLS in PWH was limited for reproducibility in children and adolescents (single study) and unknown for construct validity and responsiveness in adults and older adults.

### 3.4 TUDS

Measurement properties for TUDS were reported in two studies involving 30 PWH [29, 36], both in children and adolescents. One study reported reproducibility (*n* = 8) with a positive rating [36], and one study reported responsiveness (*n* = 9) with a positive rating [29]. Quality assessment ranged from doubtful (assessor blinding) to very good (stability of participants for evaluating responsiveness) for studies evaluating the TUDS. HJHS was not reported for either study. Overall, the level of evidence for the TUDS in PWH was limited for reproducibility and responsiveness in children and adolescents (single studies) and unknown for construct validity and responsiveness in adults and older adults.

### 3.5 30‐STS

Measurement properties for 30‐STS were reported in only one study in adults and older adults involving 17 PWH [39]. Construct validity was rated negative and responsiveness, positive. Quality assessment ranged from adequate (comparable construct) to very good (group characteristics). The median HJHS was 41, indicating the presence of considerable multi‐joint arthropathy. Overall, the level of evidence for the 30‐STS in PWH was limited for construct validity and responsiveness in adults and older adults (single studies) and unknown in children and adolescents.

## 4 Discussion

This review evaluated the reproducibility, construct validity and responsiveness properties of available performance‐based methods assessing physical function capability in PWH. We identified measurement properties for five of the seven performance‐based tests used to measure the physical function capability of PWH: TUG, 6MTW, TUDS, SLS and 30‐STS. No measurement properties were identified for SH or TS. None of the seven performance‐based tests had been tested for all measurement properties in all age categories. No test received a high grading for reproducibility, construct validity or responsiveness in PWH. Twenty‐six properties (62%) were graded as not evaluated, eight (19%) very low, six (14%) low and two (5%) moderate. The 6MWT in both age groups was the only performance‐based test graded moderate, and this was for responsiveness [29, 30, 31]. Low and very low grades were given mostly for indeterminate results, small or single studies and concerns regarding the similarity of comparator construct for construct validity.

Only the TUG in adults and older adults [15, 17, 18, 20, 21, 22, 23, 24, 25, 26] and the 6MWT in children and adolescents [27, 28, 29, 32, 33, 35, 36] have been tested for all measurement properties. The quality of evidence for the measurement properties of the 6MWT in children and adolescents was low for reproducibility [36] and construct validity [35] and moderate for responsiveness [27, 28, 29, 32, 33]. The 6MWT is a simple, low‐risk assessment that measures how far a person can walk in six minutes [43]. It is used to evaluate a person's exercise capacity, aerobic endurance, and functional ability and has been shown to predict physical fitness in healthy children and those recovering from cancer [44, 45]. Its established use in other health conditions and with evidence of a moderate rating for responsiveness in the current review, the 6MWT may be useful for evaluating the effectiveness of treatments in PWH. Although we were not able to demonstrate sufficient evidence for reproducibility or construct validity in PWH, these properties have been demonstrated in people with osteoarthritis and elderly people [44, 45, 46, 47].

The quality of evidence for measurement properties of the TUG in adults and older adults was graded very low, with no evidence of test‐retest repeatability, and conflicting findings for construct validity [17, 18, 19, 20, 23, 24, 25, 26] and responsiveness [15, 16, 17, 18, 19, 20, 21]. The TUG test measures how quickly someone can rise from a chair, walk, turn, walk back, and sit down again. It is often used to assess mobility and fall risk, most commonly in older people [48]. Although we were not able to demonstrate sufficient evidence for reproducibility in PWH, this has been demonstrated in elderly people and for patients followed up after total knee and hip arthroplasty [49, 50, 51].

There is a clear gap for all performance tests in reporting reproducibility, including persons with mild haemophilia, those with low HJHS scores and no or minimal signs of joint arthropathy. Forty‐eight percent of studies did not include a baseline measure of joint health, such as the HJHS, limiting the interpretation of evidence. As many patients now present with milder bleeding frequency and reduced arthropathy, understanding the measurement properties of core outcomes in this group is important for future management and monitoring of interventions [2]. Heterogeneity among studies limited the interpretation of evidence in this review. When studies vary in their inclusion criteria (e.g., single versus multiple affected joints, differing levels of haemophilia severity, presence/absence of arthropathy), the findings of any single study, or even a subset of studies, may not be generalisable to the broader population of individuals with haemophilia. For example, studies focusing on more severe cases where the impact of interventions on established arthropathy is more readily observable may have led to overestimation in responsiveness. Similarly, studies focusing on specific joint issues (e.g., a single severely affected ankle) as opposed to looking at overall joint health across multiple joints observed ‘performance’ or ‘outcome’ might be measuring different constructs, making it hard to compare performance outcomes across studies. Execution of performance‐based tests differ if carried out using different protocols. For example, the length of the walkway for the 6MWT (ranging from 10 to 50 m) will influence the distance walked—shorter walkways will result in a higher number of turns, reducing the total distance walked, making comparisons between outcome values impossible [52, 53]. An adequate description of the performance‐based test was not fully described in most studies; information about the performance of the test with or without shoes and the use of single or multiple trials were the most common descriptors lacking. Chair height and whether patients were able to use their arms during the TUG were not reported in any of the studies. [54] Similarly, the SLS was performed over a range of time restrictions ranging from 30‐s to unlimited. Step height was not reported for studies reporting the TUDS test. Furthermore, assessor training and the profession of the assessor was rarely reported. In a rare condition like haemophilia where large studies are challenging, synthesising findings from multiple studies is important to inform evidence‐based care. To interpret future findings from multiple studies, agreed standardisations of test performance are recommended.

Historically, outcome assessments in haemophilia focused on the body structure and function domain of the ICF, for example, HJHS [55], MRI [56], ultrasound [57, 58], with PROM assessments of activities and participation, for example, HAL [26]. Recommendations for assessment of musculoskeletal health in persons with haemophilia advocate the comprehensive framework of the International Classification of Functioning, Disability and Health (ICF) [7]. However, direct measurement of activity defined as ‘the execution of a task or action by an individual,’ and participation, defined as ‘involvement in a life situation,’ remain under‐assessed, with PROMs being the predominant method [59].

People with haemophilia on prophylaxis now have fewer bleeds and are more physically active due to rapid medical advances [60, 61, 62]. Consequently, the HJHS, an established measure of body structure and function, has been reported to lack the ability to discriminate nuanced musculoskeletal status in this population [63, 64]. Measures of exercise capacity and physical performance, in contrast, offer greater discriminatory power [8, 23]. Therefore, outcome assessments need to evolve to effectively determine the efficacy of new treatments and provide meaningful feedback to patients.

Many performance measures, however, aren't easily performed in routine clinical practice due to constraints in time, space, and equipment [8], which can affect their clinical utility. The evolution of medical care demands new outcome assessments, and performance measures of activity and participation offer a promising solution.

While our review identified seven performance‐based tests, the TS and SH were largely excluded due to a lack of studies meeting our inclusion criteria. This highlights a significant gap in the literature; these tests, commonly used in other musculoskeletal populations for balance and power assessment, remain under‐researched in haemophilia. Understanding why these potentially valuable measures are not widely studied and exploring their potential role in a comprehensive haemophilia assessment is crucial.

Our systematic review has some limitations. We have applied established standardised criteria to evaluate and rate the quality of evidence and measurement properties of performance‐based tests [9, 10, 11, 12]. These criteria may be interpreted as strict, hence the low number of studies identified, but we wanted to ensure our conclusions and recommendations were evidence‐based. Our systematic approach utilising standardised criteria and multiple blinded reviewers of evidence at each stage of the process is a strength of our review.

More evidence might be available in the literature that could be used to determine the reproducibility, construct validity or responsiveness of the methods, for example, studies that did not report between group effect sizes or confidence intervals and studies lacking theoretically derived comparable constructs. Furthermore, we included only English‐language publications and therefore may have missed some publications on measurement properties. We did not include conference abstracts due to the lack of formal peer review. We did not evaluate criterion or predictive validity, interpretability, feasibility, practicality, or floor and ceiling effects. Feasibility and practicality of the seven performance‐based methods were evaluated in our previous consensus DELPHI study [8]. Some of the performance‐based tests have been assessed for their measurement properties in healthy populations or other patient groups [65]. However, these studies were excluded, as the measurement properties of an instrument are influenced by the specific setting and population being evaluated, and consequently, the findings from these studies may not be applicable to PWH.

## 5 Conclusion

Our review highlights a growing interest in the use of performance‐based methods in evaluating physical health in PWH, with 72% of included studies published in the last 4 years, and almost half (44%) in the last 2 years. The ICF framework provides a holistic perspective on health by emphasising the interplay between an individual's abilities, activities, and the environments in which they live [5]. By assessing both performance and capacity, clinicians and researchers can gain valuable insights into a person's actual and potential capabilities. Combined with physical examination, imaging and patient experience, this person‐centred approach not only enriches an understanding of one's health but also informs targeted interventions to enhance well‐being and promote participation across diverse contexts [3].

With the currently available evidence, together with limited data in a wide range of ages and joint disease, it is not possible at this stage to recommend a core set of performance‐based methods for evaluating physical function capacity in PWH. With the increasing use of performance‐based methods, studies evaluating and confirming the measurement properties of these outcomes are a priority. Where studies aim to generate this evidence, we recommend inclusion of baseline joint health data, standardisation and clear descriptions of test methods, training and profession of those assessing performance to enable synthesis of this work. Until the evidence on measurement properties of performance‐based methods of physical function capacity is generated, we can only advocate the use of the 6MWT to monitor responsiveness to treatment in PWH. Due to the lack of studies reporting HJHS, this may only be responsive in those who are affected with arthropathy. While the 6MWT is identified as the most reliable test, future research is crucial to provide clinicians with practical guidance on interpreting these results in real‐world haemophilia management. To enhance clinical utility, studies should focus on developing interpretative frameworks that assist clinicians in applying these results within diverse haemophilia populations, considering age‐specific norms, severity levels, and their alignment with established WFH guidelines, particularly addressing the lack of performance and capacity aspects of the activities domain of the ICF.

## Author Contributions

M.B., W.D., C.H., H.H., R.M., S.P.A., F.S., K.S., M.T. and D.S. conceptualized and designed the study, developed the search strategy, screened titles, abstracts, and full text, contributed to data synthesis and interpretation of findings, and critically reviewed and revised the manuscript. M.B. and D.S. performed data extraction and assessed the risk of bias of included studies, including resolving disagreements.

## Ethics Statement

The authors have nothing to report.

## Conflicts of Interest

The authors declare no conflicts of interest.

## Supporting information




**Supporting File 1**: Search Strategy.

**Supporting File 2**: Title, abstract and full text screening flowchart.

**Supporting File 3**: PRISMA Reporting Checklist.

## Data Availability

The data that support the findings of this study are available from the corresponding author upon reasonable request. The PRISMA Reporting Checklist is attached as a Supplementary File 3.
