# Supplementary material for: Measuring Physical Function Capacity in Persons With Haemophilia: A Systematic Review of Performance‐Based Methods
Source: Haemophilia. 2025 Jul 20;31(5):840–64. doi: 10.1111/hae.70081 (PMC12462580; doi:10.1111/hae.70081)
Supplement: Supplementary file 1 — Supporting File 1: Search Strategy. [file HAE-31-840-s003.docx]

| Search number |  | Search phrase |
| --- | --- | --- |
| **CINAHL via Ebsco** | | |
| S1 | Population | (MH "Hemophilia+") OR TI (H#emophilia OR “factor VIII deficiency” OR “factor VIII disorder OR “factor 8 deficiency” OR “factor 8 disorder” OR “factor IX deficiency” OR “factor IX disorder” OR “factor 9 deficiency” OR “factor 9 disorder”) OR AB (H#emophilia OR “factor VIII deficiency” OR “factor VIII disorder OR “factor 8 deficiency” OR “factor 8 disorder” OR “factor IX deficiency” OR “factor IX disorder” OR “factor 9 deficiency” OR “factor 9 disorder”))  **Limiters** - English Language |
| S2 |  | (MH "Musculoskeletal Diseases+") OR (MH "Chronic Pain") OR (MH "Back Pain+") OR (MH "Arthralgia+") OR (MH "Neuralgia+") OR (MH "Nociceptive Pain+") OR (MH "Musculoskeletal Pain") OR (MH "Muscle Pain") OR (MH “Pain”) OR TI (“Musculoskeletal condition#” OR “musculoskeletal disorder#” OR “musculoskeletal disease#” OR “musculoskeletal injur*” OR “musculoskeletal pain” OR “lower extremity injur*” OR “leg injur*” OR MSD) OR AB (“Musculoskeletal condition#” OR “musculoskeletal disorder#” OR “musculoskeletal disease#” OR “musculoskeletal injur*” OR “musculoskeletal pain” OR “lower extremity injur*” OR “leg injur*” OR MSD) OR TI (“Knee pain” OR “knee injur*” OR “knee condition” OR “Hip pain” OR “hip injur*” OR “hip condition” OR “Ankle pain” OR “ankle injur*” OR “ankle condition” OR “foot pain” OR “foot injur*” OR “foot condition” or “leg pain” or “limb pain” OR LBP OR “back pain” or "spinal pain") OR AB (“Knee pain” OR “knee injur*” OR “knee condition” OR “Hip pain” OR “hip injur*” OR “hip condition” OR “Ankle pain” OR “ankle injur*” OR “ankle condition” OR “foot pain” OR “foot injur*” OR “foot condition” or “leg pain” or “limb pain” OR LBP OR “back pain” or "spinal pain") OR TI (“joint pain” OR arthralgia OR “Muscle pain” OR "persistent pain" OR “chronic pain” OR “arthritis pain” or “inflammatory pain” OR Neuralgia OR myalgia OR "Nociceptive Pain”) OR AB (“joint pain” OR arthralgia OR “Muscle pain” OR "persistent pain" OR “inflammatory pain” or “chronic pain” OR “arthritis pain” OR Neuralgia OR myalgia "Nociceptive Pain”) OR TI (arthritis OR arthropathy OR arthroplasty OR fracture OR "ligament reconstruction" OR osteoarthritis OR fibromyalgia OR polymyalgia OR dislocation OR tendinitis OR tendinopathy OR "joint disease" OR hypermobility) OR AB (arthritis OR arthropathy OR arthroplasty OR fracture OR "ligament reconstruction" OR osteoarthritis OR fibromyalgia OR polymyalgia OR dislocation OR tendinitis OR tendinopathy OR "joint disease" OR hypermobility)  **Limiters** - English Language |
| S3 |  | S1 OR S2 |
| S4 | Outcome  measures | TI ((6-min* N4 walk*) OR (six-min* N4 walk*) OR 6-MWT OR 6-MWD OR S-MWT OR S-MWD) OR AB ((6-min* N4 walk*) OR (six-min* N4 walk*) OR 6-MWT OR 6-MWD OR S-MWT OR S-MWD)  **Limiters** - English Language |
| S5 |  | TI (“Timed up and down stairs” OR “Timed up-down stairs” OR TUDS OR “stair test” OR “stair-climb* test” OR SCT OR “stair ascen*-descen* test” OR "stair ascen*/descen* test” OR “stairs ascen* and descen* test”) OR AB (“Timed up and down stairs” OR “Timed up-down stairs” OR TUDS OR “stair test” OR “stair-climb* test” OR SCT OR “stair ascen*-descen* test” OR "stair ascen*/descen* test” OR “stairs ascen* and descen* test”)  **Limiters** - English Language |
| S6 |  | TI (“Tandem stance” OR “Tandem stand*” OR “sharpened Romberg” OR “tandem romberg” OR “tandem balance”) OR AB (“Tandem stance” OR “Tandem stand*” OR “sharpened Romberg” OR “tandem romberg” OR “tandem balance”)  **Limiters** - English Language |
| S7 |  | TI (“One-leg* balance” OR “1-leg* balance” OR “Single-leg* balance” OR “One-leg* stand*” OR “1-leg* stand*” OR “Single-leg* stand” OR “One-leg* stance” OR “1-leg* stance” OR “Single-leg* stance” OR “unipedal stance” OR UPST) OR AB (“One-leg* balance” OR “1-leg* balance” OR “Single-leg* balance” OR “One-leg* stand*” OR “1-leg* stand*” OR “Single-leg* stand” OR “One-leg* stance” OR “1-leg* stance” OR “Single-leg* stance” OR “unipedal stance” OR UPST)  **Limiters** - English Language |
| S8 |  | TI ((Sit-to-stand N2 30-s*) or (Chair-stand N2 30-s*) or (Sit-to-stand N2 30s*) or (Chair-stand N2 30s*) or (STS N2 30-s*) or (STS N2 30s*) or (STST N2 30-s*) or (STST N2 30s*) or 30STS or 30-STS or 30sSTS or 30-STST or 30STST or 30s-STST or 30SCS or 30-CST or 30s-CST or 30CST) OR AB ((Sit-to-stand N2 30-s*) or (Chair-stand N2 30-s*) or (Sit-to-stand N2 30s*) or (Chair-stand N2 30s*) or (STS N2 30-s*) or (STS N2 30s*) or (STST N2 30-s*) or (STST N2 30s*) or 30STS or 30-STS or 30sSTS or 30-STST or 30STST or 30s-STST or 30SCS or 30-CST or 30s-CST or 30CST)  **Limiters** - English Language |
| S9 |  | TI (“Timed-up-and-go” OR "Timed-up-&-go" OR “Get-up-and-go” OR “Get-up & go” OR TUG OR GUG OR TUGT OR GUGT OR TGUGT OR 8-foot-up-and-go OR 8-feet-up-and-go OR 8foot-up-and-go OR 8feet-up-and-go OR 8-ft-up-and-go OR 8ft-up-and-go OR 8-foot-up-&-go OR 8-feet-up-&-go OR 8foot-up-&-go OR 8feet-up-&-go OR 8-ft-up-&-go OR 8ft-up-&-go OR 8UG OR 8-FUG OR 8-FUGT) OR AB (“Timed-up-and-go” OR "Timed-up-&-go" OR “Get-up-and-go” OR “Get-up & go” OR TUG OR GUG OR TUGT OR GUGT OR TGUGT OR 8-foot-up-and-go OR 8-feet-up-and-go OR 8foot-up-and-go OR 8feet-up-and-go OR 8-ft-up-and-go OR 8ft-up-and-go OR 8-foot-up-&-go OR 8-feet-up-&-go OR 8foot-up-&-go OR 8feet-up-&-go OR 8-ft-up-&-go OR 8ft-up-&-go OR 8UG OR 8-FUG OR 8-FUGT)  **Limiters** - English Language |
| S10 |  | TI ("one-leg* hop test" or "1-leg* hop test" or "single-leg* hop test" or "unilateral hop test" OR “hop for distance” OR “horizontal hop test” OR “Single-leg forward hop” OR “One-leg forward hop” OR “1-leg forward hop” OR “forward hop distance” OR “unilateral horizontal jump” OR SLH OR SLHD OR SLHT) OR AB ("one-leg* hop test" or "1-leg* hop test" or "single-leg* hop test" or "unilateral hop test" OR “hop for distance” OR “horizontal hop test” OR “Single-leg forward hop” OR “One-leg forward hop” OR “1-leg forward hop” OR “forward hop distance” OR “unilateral horizontal jump” OR SLH OR SLHD OR SLHT)  **Limiters** - English Language |
| S11 |  | S4 OR S5 OR S6 OR S7 OR S8 OR S9 OR S10 |
| S12 | Measurement properties | (MH “measurement issues and assessments+”) OR (MH “health status indicators”) OR (MH "Factor Analysis") OR (MM "Instrument Validation") OR (MM “outcome assessment”) OR (MM "Kappa Statistic") OR (MM "Discriminant Analysis")  OR  TI (psychometr* OR clinimetr* OR clinometr* OR “outcome assessment” OR “outcome measure*” OR “observer variation” OR reproducib* OR reliab* OR unreliab* OR valid* OR “coefficient of variation” OR coefficient OR homogeneity OR homogeneous OR “internal consistency” OR (Cronbach* AND alpha) OR (Cronbach* AND alphas) OR (item AND (correlation* OR selection* OR reduction*)) OR agreement OR precision OR imprecision OR “precise values” OR test-retest OR (test AND retest) OR stability OR interrater OR inter-rater OR intrarater OR intra-rater OR intertester OR inter-tester OR intratester OR intra-tester OR interobserver OR inter-observer OR intraobserver OR intra-observer OR intertechnician OR inter-technician OR intratechnician OR intra-technician OR interexaminer OR inter-examiner OR intraexaminer OR intra-examiner OR interassay OR inter-assay OR intraassay OR intra-assay OR interindividual OR inter-individual OR intraindividual OR intra-individual OR interparticipant OR inter-participant OR intraparticipant OR intra-participant OR kappa OR kappa’s OR kappas OR repeatab* OR ((replicab* OR repeated) AND (measure OR measures OR findings OR result# OR test#)) OR generaliza* OR generalisa* OR concordance OR (intraclass AND correlation*) OR discriminative OR “known group” OR “factor analysis” OR “factor analyses” OR “factor structure” OR “factor structures” OR dimension* OR subscale* OR (multitrait AND scaling AND (analysis OR analyses)) OR  “item discriminant” OR “interscale correlation*” OR error OR errors OR “individual variability” OR “interval variability” OR “rate variability” OR (variability AND (analysis OR values)) OR (uncertainty AND (measurement OR measuring)) OR “standard error of measurement” OR sensitiv* OR responsive* OR (limit AND detection) OR “minimal detectable concentration” OR interpretab* OR ((minimal OR minimally OR clinical OR clinically) AND (important OR significant OR detectable) AND (change OR difference)) OR (small* AND (real OR detectable) AND (change OR difference)) OR “meaningful change” OR “ceiling effect” OR “floor effect” OR “Item response model” OR IRT OR Rasch OR “Differential item functioning” OR DIF OR “computer adaptive testing” OR “item bank” OR “cross-cultural equivalence”)  OR  AB (psychometr* OR clinimetr* OR clinometr* OR “outcome assessment” OR “outcome measure*” OR “observer variation” OR reproducib* OR reliab* OR unreliab* OR valid* OR “coefficient of variation” OR coefficient OR homogeneity OR homogeneous OR “internal consistency” OR (Cronbach* AND alpha) OR (Cronbach* AND alphas) OR (item AND (correlation* OR selection* OR reduction*)) OR agreement OR precision OR imprecision OR “precise values” OR test-retest OR (test AND retest) OR stability OR interrater OR inter-rater OR intrarater OR intra-rater OR intertester OR inter-tester OR intratester OR intra-tester OR interobserver OR inter-observer OR intraobserver OR intra-observer OR intertechnician OR inter-technician OR intratechnician OR intra-technician OR interexaminer OR inter-examiner OR intraexaminer OR intra-examiner OR interassay OR inter-assay OR intraassay OR intra-assay OR interindividual OR inter-individual OR intraindividual OR intra-individual OR interparticipant OR inter-participant OR intraparticipant OR intra-participant OR kappa OR kappa’s OR kappas OR repeatab* OR ((replicab* OR repeated) AND (measure OR measures OR findings OR result# OR test#)) OR generaliza* OR generalisa* OR concordance OR (intraclass AND correlation*) OR discriminative OR “known group” OR “factor analysis” OR “factor analyses” OR “factor structure” OR “factor structures” OR dimension* OR subscale* OR (multitrait AND scaling AND (analysis OR analyses)) OR  “item discriminant” OR “interscale correlation*” OR error OR errors OR “individual variability” OR “interval variability” OR “rate variability” OR (variability AND (analysis OR values)) OR (uncertainty AND (measurement OR measuring)) OR “standard error of measurement” OR sensitiv* OR responsive* OR (limit AND detection) OR “minimal detectable concentration” OR interpretab* OR ((minimal OR minimally OR clinical OR clinically) AND (important OR significant OR detectable) AND (change OR difference)) OR (small* AND (real OR detectable) AND (change OR difference)) OR “meaningful change” OR “ceiling effect” OR “floor effect” OR “Item response model” OR IRT OR Rasch OR “Differential item functioning” OR DIF OR “computer adaptive testing” OR “item bank” OR “cross-cultural equivalence”)  OR  TI (G‐theory OR "G theory" OR "generalizability theory" OR "generalisability theory") OR AB (G‐theory OR "G theory" OR "generalizability theory" OR "generalisability theory")  **Limiters** - English Language |
| S13 |  | S3 AND S11 AND S12 |
| S14 |  | PT (biography OR “case study” OR directories OR interview OR editorial OR “legal cases” OR letter OR “practice guidelines” OR “Consumer/patient teaching materials” OR “Teaching materials”) NOT (MH "vertebrates+" NOT MM "human+")  **Limiters** - English Language |
| S15 |  | S13 NOT S14 |
| **Embase AND Emcare via OVID** | | |
| S16 | Population | Exp hemophilia A/ or exp hemophilia/ or exp hemophilia B/ or ("h?emophilia" or "factor 8 deficiency" or "factor 8 disorder" or "factor VIII deficiency" or "factor VIII disorder" or "factor 9 deficiency" or "factor 9 disorder" or "factor IX deficiency" or "factor IX disorder").tw,kw. |
| S17 |  | exp musculoskeletal disease/ or pain/ or exp chronic pain/ or exp inflammatory pain/ or exp limb pain/ or exp musculoskeletal pain/ or exp myalgia/ or exp neuralgia/ or exp nociceptive pain/ or exp spinal pain/ or ("Musculoskeletal condition?" or "musculoskeletal disorder?" or "musculoskeletal disease?" or "musculoskeletal pain" or "musculoskeletal injur*" or "lower extremity injur*" or "leg injur*" or MSD).tw,kw. or ("Knee pain" or "knee injur*" or "knee condition" or "Hip pain" or "hip injur*" or "hip condition" or "Ankle pain" or "ankle injur*" or "ankle condition" or "foot pain" or "foot injur*" or "foot condition" or "leg pain" or "limb pain" or "back pain" or "spinal pain LBP").tw,kw. or ("joint pain" or arthralgia or "Muscle pain" or "chronic pain" or "persistent pain" or "arthritis pain" or "inflammatory pain" or neuralgia or myalgia or "nociceptive pain").tw,kw. or (arthritis or arthropathy or arthroplasty or fracture or "ligament reconstruction" or osteoarthritis or fibromyalgia or polymyalgia or dislocation or tendinitis or tendinopathy or "joint disease" or hypermobility).tw,kw. |
| S18 |  | S16 OR S17 |
| S19 | Outcome  Measures | exp six minute walk test/ or ((6-min* adj4 walk*) or (six-min* adj4 walk*) or 6-MWT or 6-MWD or S-MWT or S-MWD).tw,kw. |
| S20 |  | ("Timed up and down stairs" or “timed up-down stairs” or TUDS or "stair test" or "stair-climb* test" or “stair ascen*-descen* test” or “stair ascen*/descen* test” or “stair* ascen* and descen* test”).tw,kw. |
| S21 |  | ("Tandem stance" or "Tandem stand*" or "sharpened Romberg" or “tandem romberg” or “tandem balance”).tw,kw |
| S22 |  | ("One-leg* balance" or "1-leg* balance" or "Single-leg* balance" or "One-leg* stand*" or "1-leg* stand*" or "Single-leg* stand" or "One-leg* stance" or "1-leg* stance" or "Single-leg* stance" or "unipedal stance" or UPST).tw,kw. |
| S23 |  | ("Sit-to-stand adj2 30-s*" or "Chair-stand adj2 30-s*" or "Sit-to-stand adj2 30s*" or "Chair-stand adj2 30s*" or "STS adj3 30-s*" or "STS adj3 30s*" or "STST adj3 30-s*" or "STST adj3 30s*" or 30STS or 30-STS or 30sSTS or 30-STST or 30STST or 30s-STST or 30SCS or 30-CST or 30s-CST or 30CST).tw,kw. |
| S24 |  | ("Timed-up-and-go" or "timed-up-&-go" or "Get-up-and-go" or TUG or GUG or TUGT or GUGT or TGUGT or 8-foot-up-and-go or 8-feet-up-and-go or 8foot-up-and-go or 8feet-up-and-go or 8-ft-up-and-go or 8ft-up-and-go or 8-foot-up-&-go or 8-feet-up-&-go or 8foot-up-&-go or 8feet-up-&-go or 8-ft-up-&-go or 8ft-up-&-go or 8UG or 8-FUG or 8-FUGT).tw,kw. |
| S25 |  | ("one-leg* hop test" or "1-leg* hop test" or "single-leg* hop test" or "unilateral hop test" OR “hop for distance” OR “horizontal hop test” OR “Single-leg forward hop” OR “One-leg forward hop” OR “1-leg forward hop” OR “forward hop distance” OR “unilateral horizontal jump” OR SLH OR SLHT OR SLHD).tw,kw |
| S26 |  | S19 OR S20 OR S21 OR S22 OR S23 OR S25 |
| S27 | Measurement properties | (instrumentation or methods or ("validation study" or "comparative study")).mp. or exp Psychometrics/ or (psychometr* or clinimetr* or clinometr*).mp. or exp "Outcome Assessment, Health Care"/ or "outcome assessment".tw. or "outcome measure*".mp. or exp "Observer Variation"/ or "observer variation".tw. or exp "Health Status Indicators"/ or exp "Reproducibility of Results"/ or exp "Discriminant Analysis"/ or (reproducib* or reliab* or unreliab* or valid* or "coefficient of variation" or coefficient or homogeneity or homogeneous or "internal consistency").tw. or (cronbach* and (alpha or alphas)).tw. or (item and (correlation* or selection* or reduction*)).tw. or (agreement or precision or imprecision or "precise values" or test-retest).mp. or (test and retest).tw. or (reliab* and (test or retest)).tw. or stability.tw. or (interrater or inter-rater or intrarater or intra-rater or (intertester or inter-tester or intratester or intra-tester) or (interobserver or inter-observer or intraobserver or intra-observer) or (intertechnician or inter-technician or intratechnician or intra-technician) or (interexaminer or inter-examiner or intraexaminer or intra-examiner) or (interassay or inter-assay or intraassay or intra-assay) or (interindividual or inter-individual or intraindividual or intra-individual) or (interparticipant or inter-participant or intraparticipant or intra-participant)).tw. or (kappa or kappas).tw. or repeatab*.mp. or ((replicab* or repeated) and (measure or measures or findings or result or results or test or tests)).mp. or (generaliza* or generalisa* or concordance or (intraclass and correlation*) or (discriminative or "known group")).tw. or ("factor analysis" or "factor analyses" or "factor structure" or "factor structures" or (dimension* or subscale*) or (multitrait and scaling and (analysis or analyses))).tw. or ("item discriminant" or "interscale correlation*" or (error or errors) or ("individual variability" or "interval variability" or "rate variability") or (variability and (analysis or values)) or (uncertainty and (measurement or measuring))).tw. or ("standard error of measurement" or sensitiv* or responsive* or (limit and detection) or "minimal detectable concentration" or interpretab*).tw. or (((minimal or minimally or clinical or clinically) and (important or significant or detectable) and (change or difference)) or (small* and (real or detectable) and (change or difference)) or ("meaningful change" or "ceiling effect" or "floor effect" or "item response model" or IRT or rasch or "differential item functioning" or DIF or "computer adaptive testing" or "item bank" or "cross-cultural equivalence")).tw. OR ("g‐theory” OR “g theory" or "generalizability theory" or "generalisability theory").tw,kw. |
| S28 |  | S18 AND S26 AND S27 |
| S29 |  | (editorial or letter).pt. not (exp animal/ not human/) |
| S30 |  | S28 NOT S29 |
| S31 |  | Limit S30 to English language |
| **Medline via OVID** | | |
| S32 | Population | exp factor xiii deficiency/ OR exp hemophilia a/ OR exp hemophilia b/ OR ("h?emophilia" or "factor 8 deficiency" or "factor 8 disorder" or "factor VIII deficiency" or "factor VIII disorder" or "factor 9 deficiency" or "factor 9 disorder" or "factor IX deficiency" or "factor IX disorder").tw,kw. |
| S33 |  | exp musculoskeletal diseases/ or exp hip injuries/ or exp leg injuries/ or exp soft tissue injuries/ or exp chronic pain/ or exp musculoskeletal pain/ or exp neuralgia/ or nociceptive pain/ or exp back pain/ or ("Musculoskeletal condition?" or "musculoskeletal disorder?" or "musculoskeletal disease?" or "musculoskeletal pain" or "musculoskeletal injur*" or "lower extremity injur*" or "leg injur*" or MSD).tw,kw. or ("Knee pain" or "knee injur*" or "knee condition" or "Hip pain" or "hip injur*" or "hip condition" or "Ankle pain" or "ankle injur*" or "ankle condition" or "foot pain" or "foot injur*" or "foot condition" or "leg pain" or "limb pain" or "back pain" or "spinal pain LBP").tw,kw. or ("joint pain" or arthralgia or "Muscle pain" or "chronic pain" or "persistent pain" or "arthritis pain" or "inflammatory pain" or neuralgia or myalgia or "nociceptive pain").tw,kw. or (arthritis or arthropathy or arthroplasty or fracture or "ligament reconstruction" or osteoarthritis or fibromyalgia or polymyalgia or dislocation or tendinitis or tendinopathy or "joint disease" or hypermobility).tw,kw. |
| S34 |  | S32 OR S33 |
| S35 | Outcome measures | exp walk test/ or ((6-min* adj4 walk*) or (six-min* adj4 walk*) or 6-MWT or 6-MWD or S-MWD or S-MWT).tw,kw. |
| S36 |  | ("Timed up and down stairs" or “timed up-down stairs” or TUDS or "stair test" or "stair-climb* test" or “stair ascen*-descen* test” or “stair ascen*/descen* test” or “stair* ascen* and descen* test”).tw,kw. |
| S37 |  | (“Tandem stance” OR “Tandem stand*” OR “sharpened Romberg” or “tandem romberg” or “tandem balance”).tw,kw. |
| S38 |  | ("One-leg* balance" or "1-leg* balance" or "Single-leg* balance" or "One-leg* stand*" or "1-leg* stand*" or "Single-leg* stand" or "One-leg* stance" or "1-leg* stance" or "Single-leg* stance" or “single-limb stance” or “unipedal stance” or UPST).tw,kw |
| S39 |  | ("Sit-to-stand adj2 30-s*" or "Chair-stand adj2 30-s*" or "Sit-to-stand adj2 30s*" or "Chair-stand adj2 30s*" or "STS adj3 30-s*" or "STS adj3 30s*" or "STST adj3 30-s*" or "STST adj3 30s*" or 30STS or 30-STS or 30sSTS or 30-STST or 30STST or 30s-STST or 30SCS or 30-CST or 30s-CST or 30CST).tw,kw. |
| S40 |  | ("Timed-up-and-go" or "timed-up-&-go" or "Get-up-and-go" or TUG or GUG or TUGT or GUGT or TGUGT or 8-foot-up-and-go or 8-feet-up-and-go or 8foot-up-and-go or 8feet-up-and-go or 8-ft-up-and-go or 8ft-up-and-go or 8-foot-up-&-go or 8-feet-up-&-go or 8foot-up-&-go or 8feet-up-&-go or 8-ft-up-&-go or 8ft-up-&-go or 8UG or 8-FUG or 8-FUGT).tw,kw. |
| S41 |  | ("one-leg* hop test" or "1-leg* hop test" or "single-leg* hop test" or "unilateral hop test" OR “hop for distance” OR “horizontal hop test” OR “Single-leg forward hop” OR “One-leg forward hop” OR “1-leg forward hop” OR “forward hop distance” OR “unilateral horizontal jump” OR SLH OR SLHT OR SLHD).tw,kw |
| S42 |  | S35 OR S36 OR S37 OR S38 OR S39 OR S40 OR S41 |
| S43 | Measurement properties | instrumentation or methods or ("validation study" or "comparative study")).mp. or exp Psychometrics/ or (psychometr* or clinimetr* or clinometr*).mp. or exp "Outcome Assessment, Health Care"/ or "outcome assessment".tw. or "outcome measure*".mp. or exp "Observer Variation"/ or "observer variation".tw. or exp "Health Status Indicators"/ or exp "Reproducibility of Results"/ or exp "Discriminant Analysis"/ or (reproducib* or reliab* or unreliab* or valid* or "coefficient of variation" or coefficient or homogeneity or homogeneous or "internal consistency").tw. or (cronbach* and (alpha or alphas)).tw. or (item and (correlation* or selection* or reduction*)).tw. or (agreement or precision or imprecision or "precise values" or test-retest).mp. or (test and retest).tw. or (reliab* and (test or retest)).tw. or stability.tw. or (interrater or inter-rater or intrarater or intra-rater or (intertester or inter-tester or intratester or intra-tester) or (interobserver or inter-observer or intraobserver or intra-observer) or (intertechnician or inter-technician or intratechnician or intra-technician) or (interexaminer or inter-examiner or intraexaminer or intra-examiner) or (interassay or inter-assay or intraassay or intra-assay) or (interindividual or inter-individual or intraindividual or intra-individual) or (interparticipant or inter-participant or intraparticipant or intra-participant)).tw. or (kappa or kappas).tw. or repeatab*.mp. or ((replicab* or repeated) and (measure or measures or findings or result or results or test or tests)).mp. or (generaliza* or generalisa* or concordance or (intraclass and correlation*) or (discriminative or "known group")).tw. or ("factor analysis" or "factor analyses" or "factor structure" or "factor structures" or (dimension* or subscale*) or (multitrait and scaling and (analysis or analyses))).tw. or ("item discriminant" or "interscale correlation*" or (error or errors) or ("individual variability" or "interval variability" or "rate variability") or (variability and (analysis or values)) or (uncertainty and (measurement or measuring))).tw. or ("standard error of measurement" or sensitiv* or responsive* or (limit and detection) or "minimal detectable concentration" or interpretab*).tw. or (((minimal or minimally or clinical or clinically) and (important or significant or detectable) and (change or difference)) or (small* and (real or detectable) and (change or difference)) or ("meaningful change" or "ceiling effect" or "floor effect" or "item response model" or IRT or rasch or "differential item functioning" or DIF or "computer adaptive testing" or "item bank" or "cross-cultural equivalence")).tw. OR ("g‐theory’OR g theory" or "generalizability theory" or "generalisability theory").tw,kw. |
| S44 |  | S34 AND S42 AND S43 |
| S45 |  | (address or autobiography or biography or "case reports" or comment or directory or editorial or festschrift or interview or lecture or "legal case" or legislation or letter or news or "newspaper article" or "patient education handout" or congress or "consensus development conference" or "consensus development conference, nih" or "practice guideline").pt. not (exp animals/ not exp humans/) |
| S46 |  | S44 NOT S45 |
| S47 |  | Limit S46 to English language |
| **Cochrane** | | |
| S48 | Population | MeSH descriptor: [Hemophilia A] this term only |
| S49 |  | MeSH descriptor: [Hemophilia B] this term only |
| S50 |  | MeSH descriptor: [Musculoskeletal Diseases] explode all trees |
| S51 |  | (“Musculoskeletal condition?” OR “musculoskeletal disorder#” OR “musculoskeletal disease?” OR “musculoskeletal injur*” OR “lower extremity injur*” OR “leg injur*” OR MSD) OR (“Knee pain” OR “knee injur*” OR “knee condition” OR “Hip pain” OR “hip injur*” OR “hip condition” OR “Ankle pain” OR “ankle injur*” OR “ankle condition” OR “foot pain” OR “foot injur*” OR “foot condition”) OR (“joint pain” OR arthralgia OR “Muscle pain” OR “chronic pain” OR “arthritis pain”) OR (arthritis OR arthropathy OR arthroplasty OR fracture OR "ligament reconstruction" OR osteoarthritis OR fibromyalgia OR polymyalgia OR dislocation OR tendinitis OR tendinopathy OR "joint disease" OR hypermobility) |
| S52 |  | S48 OR S49 OR S50 OR S51 |
| S53 | Outcome measures | (“six-min walk test” OR “6-min walk* test” OR “six-min walk distance” OR “6-min walk distance” OR 6MWT OR 6MWD OR SMWT OR SMWD) |
| S54 |  | (“Timed up and down stairs” OR “Timed up-down stairs” OR TUDS OR “stair test” OR "stair-climb test") |
| S55 |  | (“Tandem stance” OR “Tandem stand*” OR “sharpened Romberg” OR “tandem romberg” OR “tandem balance”) |
| S56 |  | ("One-leg balance" OR "1-leg balance" OR "Single-leg balance" OR "One-leg stand" OR "1-leg stand" OR "Single-leg stand" OR "One-leg stance" OR "1-leg stance" OR "Single-leg stance" OR “unipedal stance” OR UPST) |
| S57 |  | ("Sit-to-stand in 30 sec" OR "30sec sit-to-stand" OR "Chair-stand in 30 sec" OR "30 sec chair-stand”"OR "30s STS" OR 30CST) |
| S58 |  | (“Timed-up-and-go” OR "Timed-up & go" OR “Get-up-and-go” OR “Get-up & go” OR TUG OR GUG OR TGUT OR GUGT OR TGUGT) |
| S59 |  | ("one-leg hop test" or "1-leg hop test" or "single-leg hop test" or "unilateral hop test" OR "hop for distance" OR "horizontal hop test" OR "Single-leg forward hop" OR "One-leg forward hop" OR "1-leg forward hop" OR "forward hop distance" OR "unilateral horizontal jump" OR SLH OR SLHD) |
| S60 |  | S53 OR S54 OR S55 OR S56 OR S57 OR S58 OR S59 |
| S61 |  | valid* OR reliabil* OR responsiv* OR reproduc* OR "error measurement" OR “measurement error” or interrater OR intrarater or “test-retest” |
| S62 |  | S52 AND S60 AND S61 |
