# Supplementary material for: Measuring Physical Function Capacity in Persons With Haemophilia: A Systematic Review of Performance‐Based Methods
Source: Haemophilia. 2025 Jul 20;31(5):840–64. doi: 10.1111/hae.70081 (PMC12462580; doi:10.1111/hae.70081)
Supplement: Supplementary file 2 — Supporting File 2: Title, abstract and full text screening flowchart. [file HAE-31-840-s002.pdf]

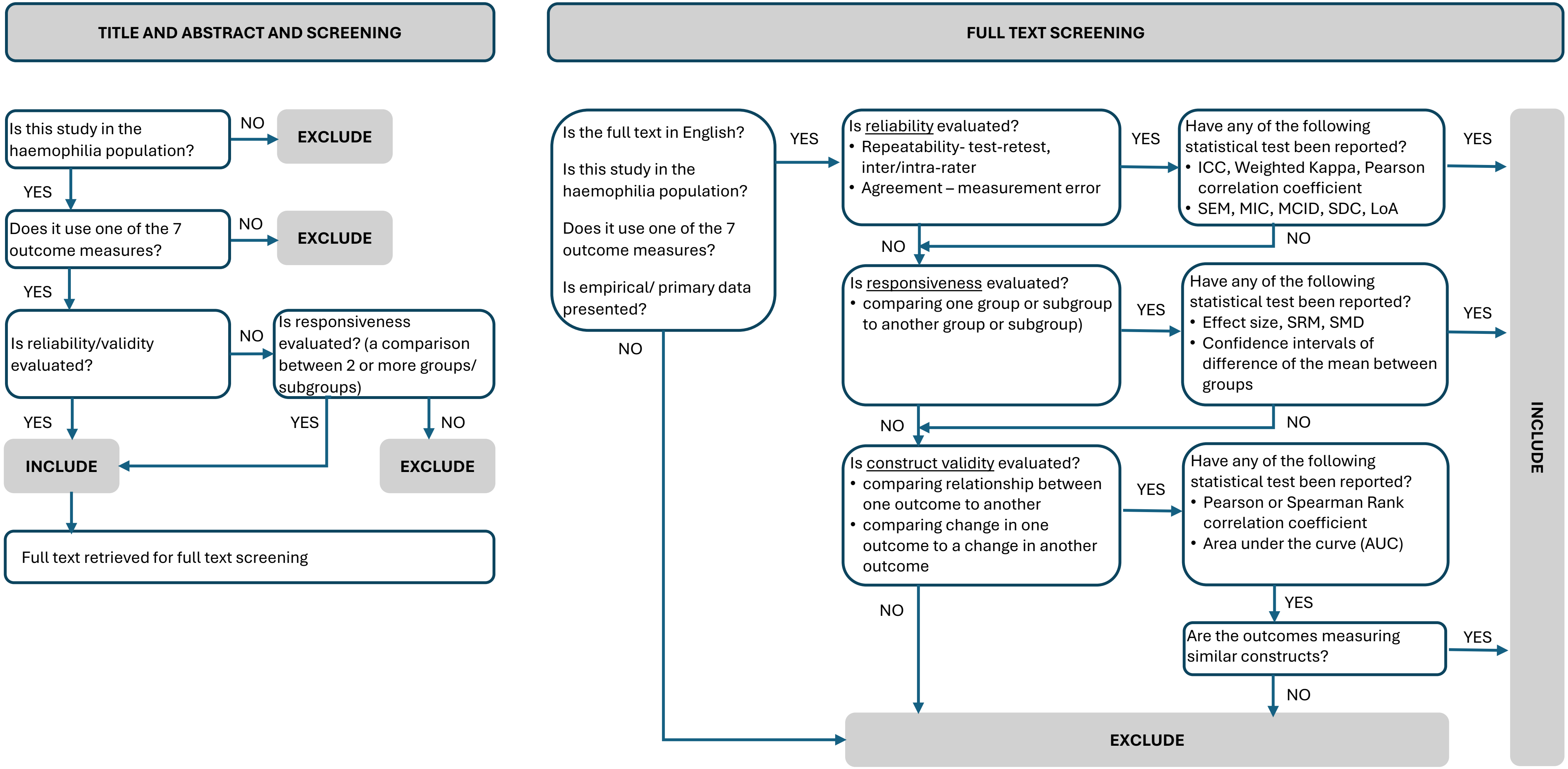

Supplementary File 2. Title, abstract and full text screening flowchart

ICC = Intraclass Correlation Coefficient; SEM = Standard Error of Measurement; MIC = Minimal Important Change; MCID = Minimal Clinically Important Difference; SDC = Smallest Detectable Change; LoA = Levels of Agreement; Standardised Response Mean; SMD = Standardised Mean Difference; AUC = Area Under the Curve
